# Effect of Volume Tie Ratio in the Engineered Cementitious Composites Plastic Hinges on the Seismic Performance of RC Composite Bridge Columns

**DOI:** 10.3390/ma14195739

**Published:** 2021-10-01

**Authors:** Qian Li, Kedao Chen, Rui Zhang, Xi Li, Wenjin Zhang

**Affiliations:** 1 Department of Bridge Engineering, School of Civil Engineering, Southwest Jiaotong University, Chengdu 610031, China; liqianchiping@outlook.com (Q.L.); chen_kedao@my.swjtu.edu.cn (K.C.); 2 Key Laboratory of High-Speed Railway Engineering, Ministry of Education, Southwest Jiaotong University, Chengdu 610031, China; 3 School of Civil Engineering, Qingdao University of Technology, Qingdao 266525, China; xi.li@qut.edu.cn; 4 Guangzhou Branch, China Huaxi Engineering Design Construction Corporation, Guangzhou 510600, China; w.jin.zhang@foxmail.com

**Keywords:** ECC, lateral confinement, volume tie ratio, bridge column, hybrid loading

## Abstract

The feasibility that the transverse reinforcements in steel-reinforced Engineered Cementitious Composites (ECC) columns could be reduced or even totally eliminated has been experimentally demonstrated. However, due to the effect of the tie volume ratio in ECC plastic hinges on the seismic performance of RC composite bridge columns not being fully clarified as of yet, a numerical study was carried out. In this study, the analytical models based on the fiber element method, by considering the superposition of different lateral confinements resulting from ties and the ECC cover, were used to correlate with a target hybrid-loading experiment. Load-displacement hysteresis, strains in extreme fibers and longitudinal bars in analytical results correlated well with the experiments, verifying the accuracy of the analytical models proposed in this study. Based on the analytical results, it was found that the volume tie ratio had little effect on the stress-strain hysteresis of the ECC cover, but a lower volume tie ratio resulted in more significant nonlinear behavior longitudinally. Finally, the pushover analysis was conducted to investigate the effect of volume tie ratios on the seismic design parameters, and the results showed that a higher volume tie ratio resulted in a limited increase in the maximum allowable displacement for design.

## 1. Introduction

Reinforced concrete (RC) bridge columns suffered extensive damage in past major seismic events, such as the 1976 Tangshan, 1994 Northridge, 1995 Kobe, 2008 Wenchuan and 2011 Tohoku earthquakes. Most of those bridge columns were damaged because of insufficient ductility, especially the insufficient ductility in plastic hinges [1]. To prevent potential shear failure and improve the ductility of bridge columns, more and more tie bars in the plastic hinge region were required by the seismic codes in many countries, so that the core concrete could be confined [2], and the buckling of longitudinal bars could be mitigated [3]. Those approaches did enhance the seismic performance of RC bridge columns in recent earthquakes with minor or even moderate intensities, but they still barely prevented the total collapse in an earthquake with high intensity due to the upper limit resulting from the brittle nature of concrete [4]. In addition, more and more densely arranged ties in the plastic hinge region of RC bridge columns increased the difficulty in placing and compacting concrete during the construction phase. It may result in placing defects and trigger future durability problems.

Engineered Cementitious Composites (ECC) [5], as one kind of emerging high-performance fiber-reinforced cementitious composite, in recent decades featured excellent tensile behavior, i.e., multiple fine cracking and metal-like strain-hardening behavior. Compared to normal strength concrete, ECC has a comparable tensile and compressive strength but a much larger tensile and compressive strain capacity [6]. In general, the ultimate tensile strain of ECC is greater than 2.0%, and the strain corresponding to the compressive strength is about twice as that of concrete. As a result, the elastic modulus of ECC is approximately half as that of normal strength concrete due to the lack of coarse aggregate [6]. The larger strain capacity and multiple cracking behavior in ECC makes it an ideal replacement of normal concrete in the plastic hinge region of RC bridge columns.

In recent decades, research on the application of ECC in the plastic hinge region of RC columns was investigated by several researchers. Fishcer et al. [7] investigated the cyclic response of a cantilever column without ties but using ECC. Results exhibited that the ductility, load-carrying capacity, and damage tolerance of ECC columns without ties were dramatically improved compared to that of RC columns with ties. Saiidi and Wang [8] proposed to use precast segments made of polyvinyl alcohol (PVA) fiber-reinforced engineered cementitious composites (PVA-ECC) with shape memory alloys (SMA) bars in the potential region of plastic hinges in bridge columns. The shake-table tests were conducted, and the results exhibited that only minor damage under strong ground motions occurred. Kawashima et al. [9] compared the seismic performance of bridge columns using different materials which were concrete, steel fiber-reinforced concrete and ECC in the region of plastic hinge. The column using ECC exhibited the best performance due to the substantial mitigation of damage in cover concrete and local buckling in longitudinal bars. Moreover, the seismic performance of a full-scaled bridge column using ECC in the plastic hinge (C1-6) was also investigated on the world’s largest shake table—E-Defense [10]. The test results showed that the implementation of ECC in the plastic hinge significantly reduced the damage and maintained the functionality of the bridge after an earthquake. In order to clarify the effect of volumetric tie ratio in ECC plastic hinges further, Zhang et al. [11] conducted the hybrid-loading tests on two scaled columns with different volumetric tie ratios based on C1-6. Experimental results showed that the column with reduced ties still exhibited a comparable seismic performance as that without the reduction in ties. In order to study the lateral confining effect further, Li et al. [12] conducted the axial compression tests on the short concrete column confined by ECC jackets and found that the lateral confining effect resulting from the tensile behavior of ECC contributed significantly to the peak strength. Cai et al. [13] studied the cyclic response of ECC-encased concrete filled steel tube (CFST) columns based on a nonlinear analysis but without consideration of the lateral confining effect from ECC.

Although those studies experimentally demonstrated the feasibility of reducing the ties in ECC plastic hinges of RC columns under seismic loading, few analytical studies have been conducted so far. Therefore, the effect of the volume tie ratio in ECC plastic hinges on the seismic performance of the RC composite column have yet to be clarified. To investigate the effects of the volume tie ratio in ECC plastic hinges on the seismic performance of RC composite bridge columns, the calculation of the lateral confining stress of the ECC cover was proposed, and the fiber-element-based model with consideration of the lateral confining effect from the ECC cover was verified by a target experiment which incorporates two scaled composite columns [11] with different volume tie ratios. Then, based on the verified numerical models, a deep study was carried out.

## 2. Target Experimental Program and Results

### 2.1. Target Experimental Program

**Figure 1:**
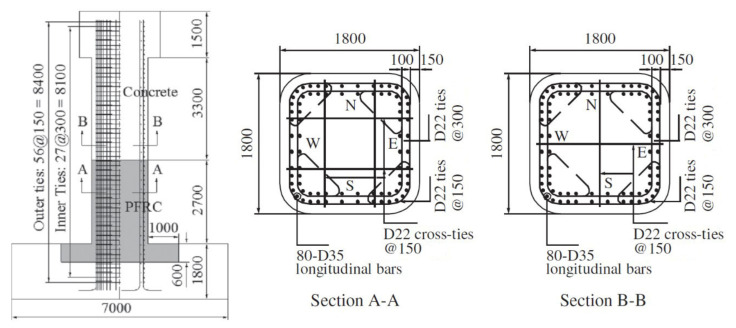
Dimension and configuration of C1-6 (mm).

The prototype of the scaled bridge columns in the target experiment [11] was the full-size bridge column incorporating ECC in the region of the plastic hinge (C1-6) [10] as mentioned above. The dimension and configuration of the prototype column are shown in Figure 1. It had a height of 7.5 m and a square cross section of 1.8 × 1.8 m^2^ and was designed in accordance with the 2002 JRA code [14]. ECC was only used in part of the column with a depth of 2.7 m above the column base and in part of the footing with a depth of 0.6 m below the column base. ECC with a designed compressive strength of 40 MPa was used in the prototype, which consisted of cement, fly ash, fine aggregate, superplasticizer, water and a 3% volume of polypropylene (PP) fibers, as tabulated in Table 1. The axial compressive and tensile strength of ECC was 43.0 MPa and 2.4 MPa, respectively [15], which was obtained by cylinder tests with a diameter of 100 mm and height of 200 mm [14]. Concrete with a designed compressive strength of 30 MPa was used, and the actual average 28-day axial compressive strength was 41 MPa [10].

**Figure 2:**
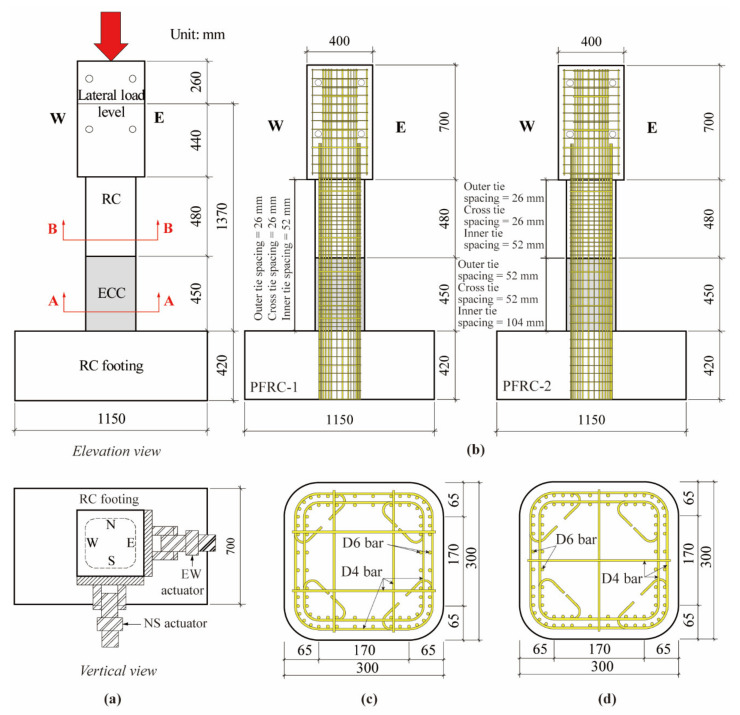
Dimension and configuration of PFRC-1 and PFRC-2. (**a**) Dimensions, (**b**) Configurations of columns, (**c**) A-A section, (**d**) B-B section.

In the target experiment [11], two scaled bridge columns using ECC at the column base with the scale ratio of 6/35 named by PFRC-1 and PFRC-2 were designed as shown in Figure 2. It should be noted that the ECC with a designed compressive strength of 40 MPa was used in the target experiment [11] which had the same mixture as that of C1-6. Except for the volume tie ratio in the ECC portion in the two columns, both columns had identical dimensions and longitudinal reinforcements as shown in Figure 2b–d. According to the scale ratio, the length similitude parameter $S_{L}$ and the displacement similitude parameter $S_{\Delta }$ were both set to 6/35. Furthermore, because the materials of the scaled bridge columns were the same as the prototype, the elastic modulus similitude parameter $S_{E}$ was set to 1. Then, based on the similitude relations given in Table 2, the stress similitude parameter $S_{\sigma }$ and the force similitude parameter $S_{F}$ could be obtained and are also presented in Table 2. The total height of the columns was 1630 mm, while the height from the column base to the lateral load level was 1370 mm. The dimension of the cross section was 300 × 300 mm^2^ with round corners. The deformed bars with the nominal diameter of 6 mm were used as two-layer longitudinal reinforcements. The longitudinal reinforcement ratio in both columns was 2.61%. The deformed bars with the nominal diameter of 4 mm were used as outer, inner and cross ties. The outer and cross ties were arranged at a spacing of 26 mm, and the inner ties were at a spacing of 52 mm in PFRC-1, while in the ECC portion of PFRC-2, they were arranged at a double spacing of PFRC-1. As for the ties in the concrete portion above and below the ECC portion, they were arranged at the same spacing in both columns. As a result, the volume tie ratio in the ECC portion of PFRC-1 was 1.51%, while it was 0.76% in PFRC-2. Based on the results of material tests which followed the JSCE code [14], the axial compressive strength measurements of ECC and concrete were 45.1 MPa and 33.7 MPa, respectively [16], which were obtained by cylinders with diameters of 100 mm and height of 200 mm.

**Figure 3:**
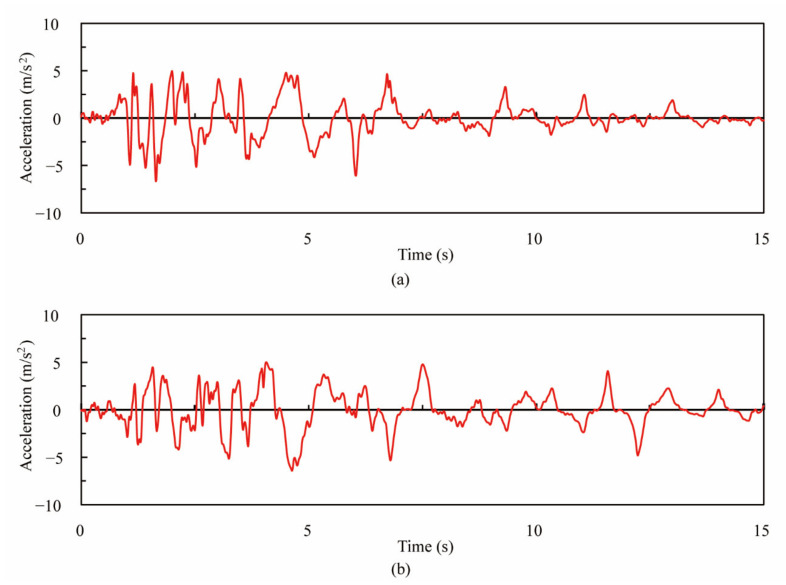
Acceleration record at JR Takatori Station during 1995 Kobe Earthquake. (**a**) EW direction, (**b**) NS direction.

The hybrid loading experiment was conducted, and the frequency of data recording was 10 Hz. The hybrid loading was provided by three hydraulic actuators in total, including one in the vertical and two in the lateral direction. Two lateral actuators applied the response displacement based on the acceleration record at the JR Takatori Station during the 1995 Kobe Earthquake (Figure 3). To apply the same compressive stress of 1 MPa as that of the prototype, one vertical actuator applied a constant uniaxial load of 86.4 kN on behalf of the dead load from superstructure on the top of the column. For one column, a total of 3 excitations in a consecutive way were applied. The intensity of ground acceleration during the 1st, 2nd and 3rd excitations was set as 10%, 20% and 25% of the original record from the response displacement of the JR Takatori ground acceleration, respectively. Moreover, the details of the experimental setup can be referred to in the target study [11].

### 2.2. Target Experimental Results

**Figure 4:**
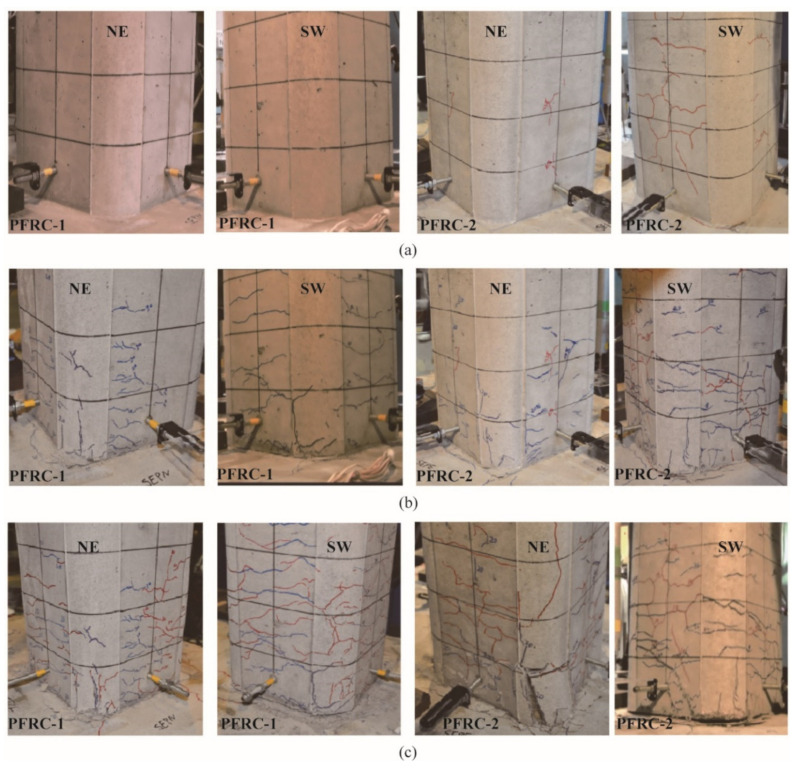
Damage development at the column base in PFRC-1 and PFRC-2. (**a**) After 1st excitation, (**b**) After 2nd excitation, (**c**) After 3rd excitation.

In the target experiments, most of the cracks were observed as less than the height of 150 mm from the column base in both columns. Due to the principal direction of the lateral response displacement being about 20 degrees east by north, most cracks adjacent to the column base in the NE and SW direction after the 1st, 2nd and 3rd excitations in PFRC-1 and PFRC-2 are shown in Figure 4. During the 1st excitation, almost no cracks developed in PFRC-1, while some cracks developed in PFRC-2. As the loading progressed into the 2nd excitation, some horizontal cracks developed in PFRC-1, and more cracks developed in PFRC-2. During the 3rd excitation, except for the development of more cracks in PFRC-1, the separation between the column base and top surface of the footing occurred as shown in Figure 4c. The damage in PFRC-2 was more significant than PFRC-1 due to the occurrence of ECC spalling at the column base (Figure 4c). However, overall, compared to the normal RC column [9], no significant damage in the region of the plastic hinge occurred in PFRC-1 or PFRC-2, which verified the feasibility of reducing ties in the ECC part.

## 3. Fiber Element-Based Analysis

### 3.1. Analytical Model

**Figure 5:**
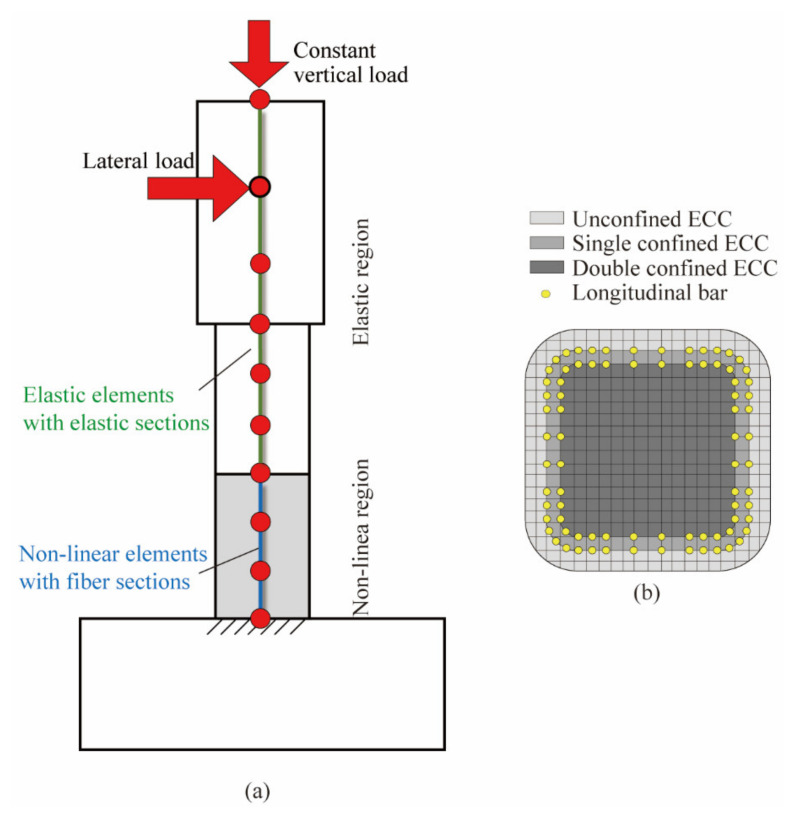
Detail of analytical model. (**a**) Fiber element model, (**b**) Division of fibers.

To clarify further the effect of the volume tie ratio in the ECC plastic hinge on the seismic performance of the RC composite columns, the fiber element models (Figure 5) considering different effects of lateral confinements based on OpenSees were established to correlate with the experimental results of PFRC-1 and PFRC-2. The elements in the non-linear region were modeled by non-linear elements of the ‘Displacement-Based Beam-Column Element’ with the ‘Fiber Section’ as defined in OpenSees, while the elements in the elastic region were modeled by elastic elements of the ‘Elastic Beam Column Element’ with the ‘Elastic Section’. The node at the column base was fixed. The constant vertical force and lateral loads were respectively defined as the ‘Constant TimeSeries’ and ‘Path TimeSeries’ and were applied at the location as shown in Figure 5a by the ‘NodalLoad pattern’ in OpenSees (Version 3.2.1, University of California, Berkeley, CA, USA). In the analysis, the superposition of the different lateral confinements resulting from the ties and ECC was considered. Figure 5b shows the division of fibers in the ECC fiber elements. The cover fibers out of the outer ties without lateral confinements were designated as ‘unconfined ECC’. The fibers out of the inner ties but within outer ties were confined by the outer ties and ECC cover and thereby designated as ‘double-confined ECC’. The core fibers within the inner ties were confined by both outer and inner ties and ECC out of the inner ties and were designated as ‘triple-confined ECC’. The properties of these fibers will be discussed in the following section.

### 3.2. Effect of Lateral Confinement

**Figure 6:**
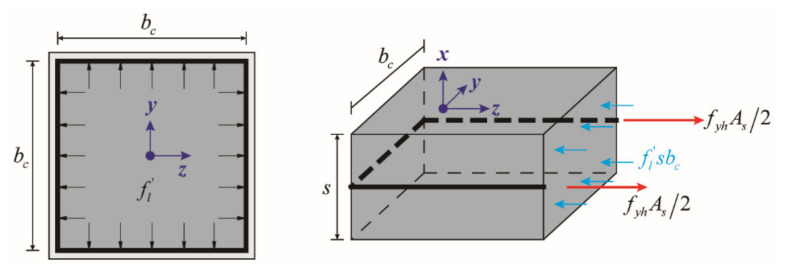
Forces acting on the free body of the confined concrete by ties.

**Figure 7:**
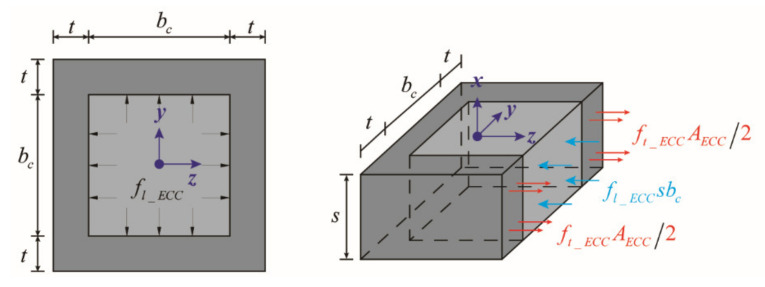
Forces acting on the free body of the core ECC confined by ECC cover.

Previous research [17,18,19] shows that the lateral confinement to the concrete provided by the ties in the RC columns results in a significant improvement in both strength and ductility. The ultimate compressive capacity of the confined concrete is achieved when the ties were on the yielding stage in tension. Based on the equilibrium of forces acting on the free body of the confined concrete by the ties as shown in Figure 6, the lateral confining stress ($f_{l}^{\prime }$) provided by the ties could be calculated by Equations (1) and (2):

(1) $$\begin{array}{c} f_{l}^{\prime }sb_{c}=f_{yh}A_{s} \end{array}$$

(2) $$\begin{array}{c} f_{l}^{\prime }=\frac{f_{yh}A_{s}}{sb_{c}} \end{array}$$

in which s is the spacing of ties in the longitudinal direction; $b_{c}$ is the width of the confined concrete; $f_{yh}$ is the yield strength of the tie bars; $A_{s}$ is the total area of the ties. Because of the arch actions due to discontinuous confinement, the effective lateral confining stress ($f_{l}$) is derived based on Equations (3) to (6) [20]:

(3) $$\begin{array}{c} f_{l}=k_{e}f_{l}^{\prime } \end{array}$$

(4) $$\begin{array}{c} k_{e}=A_{e}/A_{cc} \end{array}$$

(5) $$\begin{array}{c} A_{e}=\left(b_{c}^{2}-\overset{n}{\underset{i}{\sum }}\frac{{\left(w_{i}^{\prime }\right)}^{2}}{6}\right){\left(1-0.5\frac{s^{\prime }}{b_{c}}\right)}^{2} \end{array}$$

(6) $$\begin{array}{c} A_{cc}=b_{c}^{2}-A_{st} \end{array}$$

in which $k_{e}$ is the effective confinement factor; $A_{e}$ is the area of effectively confined concrete; $A_{cc}$ is the total area of confined concrete; $w_{i}^{\prime }$ is the clear spacing of longitudinal bars in the cross section; $s^{\prime }$ is the clear spacing of ties in the column axis. The peak strength ($f_{cc}^{\prime }$), as well as its corresponding strain ($\varepsilon_{cc}^{\prime }$) of the confined concrete, is derived based on the equations in [21] as expressed by Equations (7) and (8):

(7) $$\begin{array}{c} f_{cc}^{\prime }=f_{c}^{\prime }+k_{1}f_{1} \end{array}$$

(8) $$\begin{array}{c} \varepsilon_{cc}^{\prime }=\varepsilon_{c}^{\prime }\left(1+\frac{k_{2}f_{l}}{f_{c}^{\prime }}\right) \end{array}$$

in which $f_{c}^{\prime }$ and $\varepsilon_{c}^{\prime }$ are the peak strength and corresponding strain of the unconfined concrete, respectively; $k_{1}$ and $k_{2}$ are factors of the concrete and the lateral confining stress, respectively, and can be calculated based on equations in [2]. Since there is no constitutive model for confined ECC, the calculation of the peak strength ($f_{cc\_ECC}^{\prime }$) and corresponding strain ($\varepsilon_{cc\_ECC}^{\prime }$) of the confined ECC still employed the same method as aforementioned in Equations (1)–(8). Furthermore, it was assumed that the ECC cover would also confine the core ECC due to its excellent tensile, ductile behavior. The lateral confining stress provided by the ECC cover ($f_{l\_ECC}$) acting on the core ECC is derived based on the equilibrium of forces as shown in Figure 7 and expressed by Equation (9):

(9) $$\begin{array}{c} f_{l\_ECC}sb_{c}=f_{t\_ECC}A_{ECC} \end{array}$$

in which $f_{t\_ECC}$ is the yield strength of ECC in tension; $A_{ECC}$ is the area of ECC providing lateral confining stress at the height of the tie spacing, and it can be obtained by Equation (11):

(10) $$\begin{array}{c} f_{l\_ECC}=\frac{f_{t\_ECC}A_{ECC}}{sb_{c}} \end{array}$$

(11) $$\begin{array}{c} A_{ECC}=2\cdot t\cdot s \end{array}$$

in which t is the thickness of the ECC cover. By substituting Equation (11) into Equation (10), $f_{l\_ECC}$ is obtained as Equation (12):

(12) $$\begin{array}{c} f_{l\_ECC}=\frac{2tf_{t\_ECC}}{b_{c}} \end{array}$$

Different from the ties and longitudinal bars, ECC coveris the continuum in the lateral and longitudinal direction so that the arch action due to the discontinuity of confinement in both directions could be ignored. As a result, for the steel-reinforced ECC column, the total lateral confinement to the core ECC contains effects both from the ties ($f_{l}$) and ECC cover ($f_{l\_ECC}$), and the core ECC could be defined as the ‘double-confined ECC’. Due to the multiple fine cracking effects of ECC, an empirical reduction factor of 0.5 was induced for the purpose of being conservative. Hence, the compressive strength of the core ECC ($f_{cc\_ECC}^{\prime }$) and its corresponding strain ($\varepsilon_{cc\_ECC}^{\prime }$) of the ‘double-confined ECC’ can be obtained by Equations (13) and (14):

(13) $$\begin{array}{c} f_{cc\_ECC}^{\prime }=f_{c}^{\prime }+k_{1}\left(f_{l}+0.5f_{l\_ECC}\right) \end{array}$$

(14) $$\begin{array}{c} \varepsilon_{cc\_ECC}^{\prime }=\varepsilon_{c}^{\prime }\left(1+\frac{k_{2}\left(f_{l}+0.5f_{l\_ECC}\right)}{f_{c}^{\prime }}\right) \end{array}$$

The compressive properties of the ‘double-confined ECC’ and ‘triple-confined ECC’ can be calculated by the superposition of different lateral confinements from the ties and ECC cover.

### 3.3. Material Models

**Figure 8:**
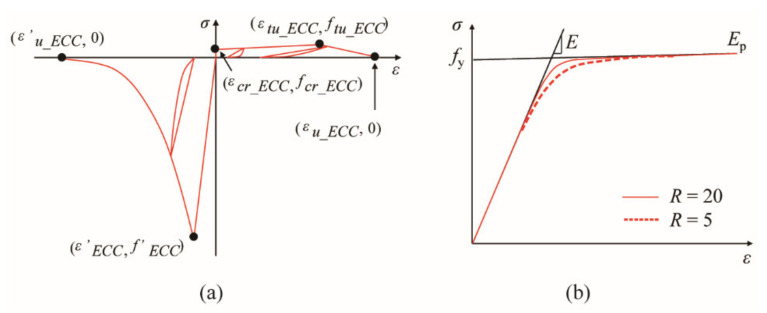
Material model. (**a**) Constitutive model for ECC, (**b**) Constitutive model for longitudinal bar.

In this study, unconfined, double-confined and triple-confined ECC were all modeled by ‘Engineered Cementitious Composites Material’ in OpenSees [22] as shown in Figure 8a. The key parameters in this model of the unconfined ECC were determined based on the experimental results of cylinder compression and uniaxial tensile tests, as specified by the JSCE code [14]. The parameters of the double-confined and triple-confined ECC model in compression were determined based on Equations (10)–(14), according to the different arrangement of the outer, inner and cross ties in the ECC portion in both columns. It should be noted that the effect of the volume tie ratio on the confined ECC in tension was neglected, and thereby the parameters of ECC in tension were identical to the material test result in the target study [11]. Consequently, the parameters of ECC at different locations in both PFRC-1 and PFRC-2 were tabulated in Table 3. The longitudinal bars were modeled by ‘Steel02’ as shown in Figure 8b, which was based on the Giuffré-Menegotto-Pinto model with isotropic strain hardening [23]. The parameters in the ‘Steel02’ model in both columns were determined based on the target experiments [11], as tabulated in Table 4.

### 3.4. Analytical Correlation Results

**Figure 9:**
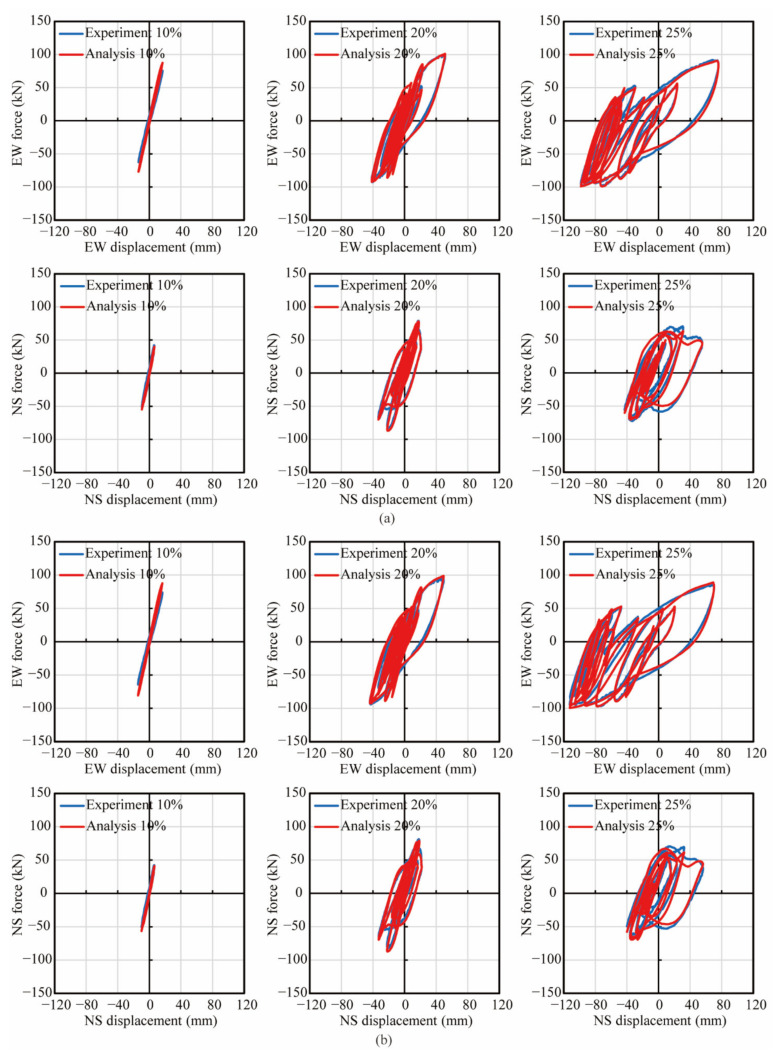
Analytical correlation of lateral forces vs. displacement with experimental results. (**a**) PFRC-1, (**b**) PFRC-2.

**Figure 10:**
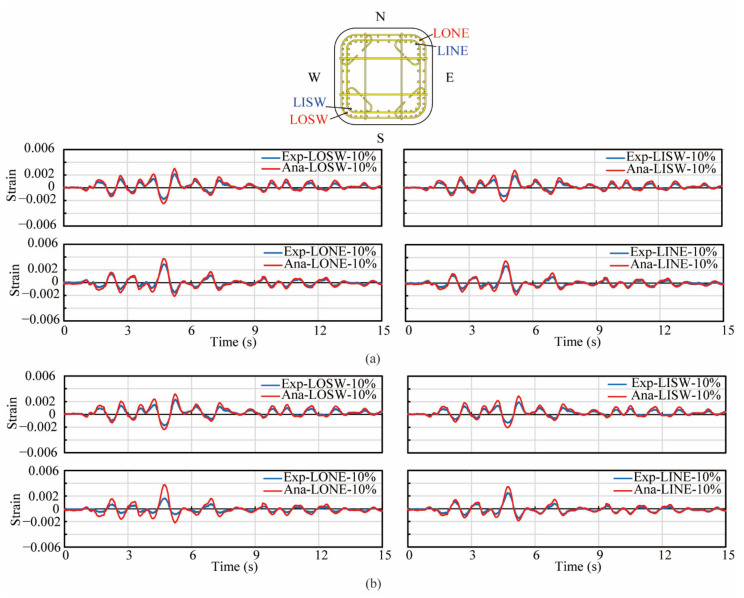
Correlation of strain in longitudinal reinforcements during the 1st excitation. (**a**) PFRC-1, (**b**) PFRC-2.

**Figure 11:**
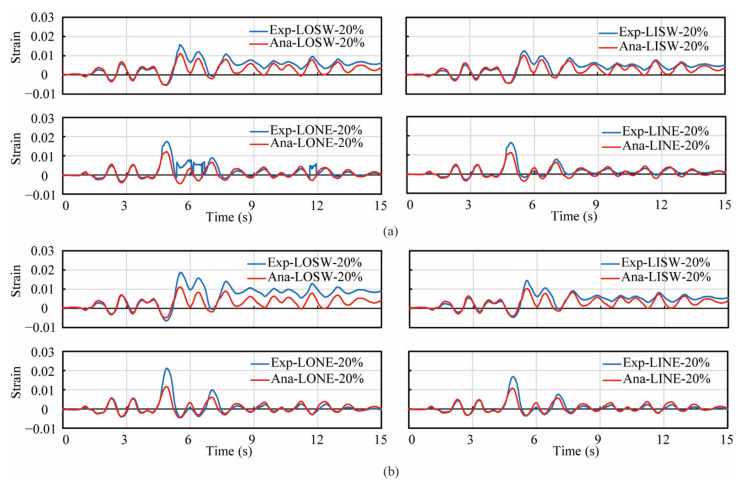
Correlation of strain in longitudinal reinforcements during the 2nd excitation. (**a**) PFRC-1, (**b**) PFRC-2.

Figure 9 shows the analytical correlation with the experimental hysteresis of lateral force vs. displacement in the EW and NS direction during three excitations in both columns. The first row of sub-figures in both Figure 9a,b shows the hysteresis of the lateral force vs. displacement during three excitations in the EW direction, while the second row shows that in the NS direction. Analytical results in both columns during the 2nd and 3rd excitations correlated well with experimental result in both the EW and NS direction. However, during the 1st excitation in the EW direction, the stiffness and peak force in the analytical results in both columns were slightly larger than those of the experiments. Additionally, the strains of the longitudinal reinforcements at the height of 104 mm during the 1st and 2nd excitations in the experiment and analytical models were compared as shown in Figure 10 and Figure 11. Since most strain gauges were damaged during the 3rd excitation, the strain data during the 3rd excitation were not compared. In the legend of Figure 10 and Figure 11, ‘Exp.’ stands for the experimental value, while ‘Ana.’ stands for the analytical value. ‘LOSW’, ‘LISW’, ‘LONE’ and ‘LINE’ stand for the ‘outer longitudinal bar in SW direction’, ‘inner longitudinal bar in SW direction’, ‘outer longitudinal bar in NE direction’ and ‘inner longitudinal bar in NE direction’, respectively, as illustrated by the sub-figure of the cross section at the top of Figure 10 and Figure 11. In Figure 10, during the 1st excitation, the stains of outer and inner longitudinal reinforcements both showed good agreement with experimental values which were obtained from the strain gauges. Besides, the outer longitudinal reinforcements were just yielded, while the inner longitudinal reinforcements were still within the elastic range. In Figure 11, during the 2nd excitation, the strains of the outer and inner longitudinal reinforcements in the analytical models correlated well with the experimental results before the 5th second, while some discrepancy occurred after the 5th second. It was considered that the strain gauges were damaged during the 2nd excitation due to these longitudinal bars undergoing large inelastic deformation more than 0.01, while the yielding strain of the steel bars was approximately 0.002.

## 4. Effect of Volume Tie Ratio

### 4.1. Hysteresis of Stress-Strain in Extreme Fibers of Cover and Core Concrete

**Figure 12:**
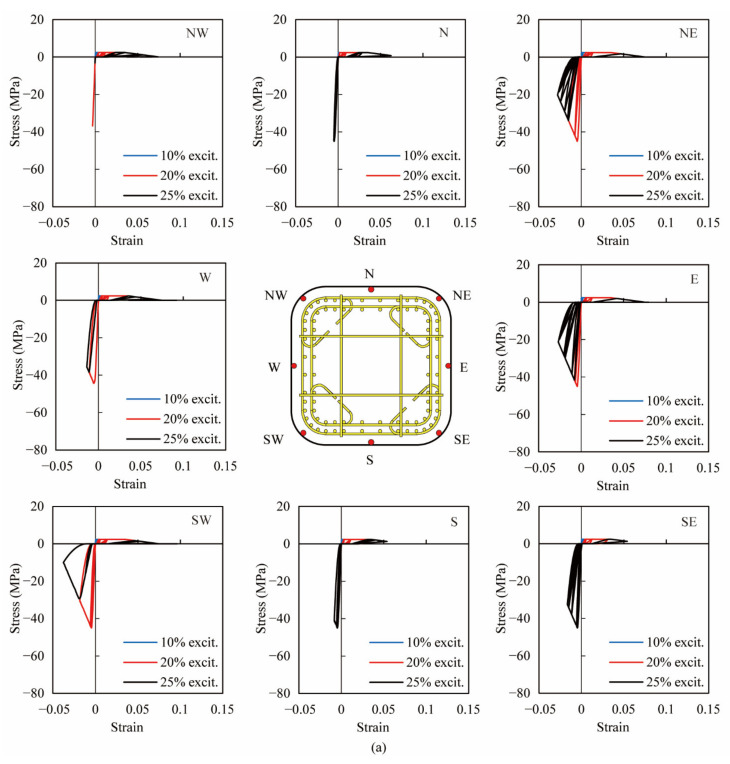

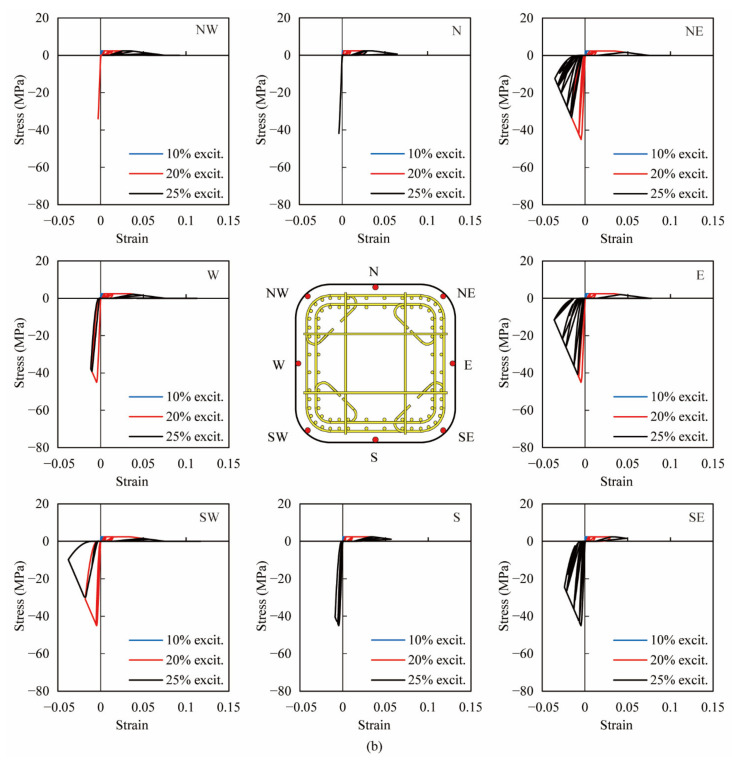
Analytical stress-strain hysteresis of extreme fibers in cover at the column base in eight directions. (**a**) PFRC-1, (**b**) PFRC-2.

**Figure 13:**
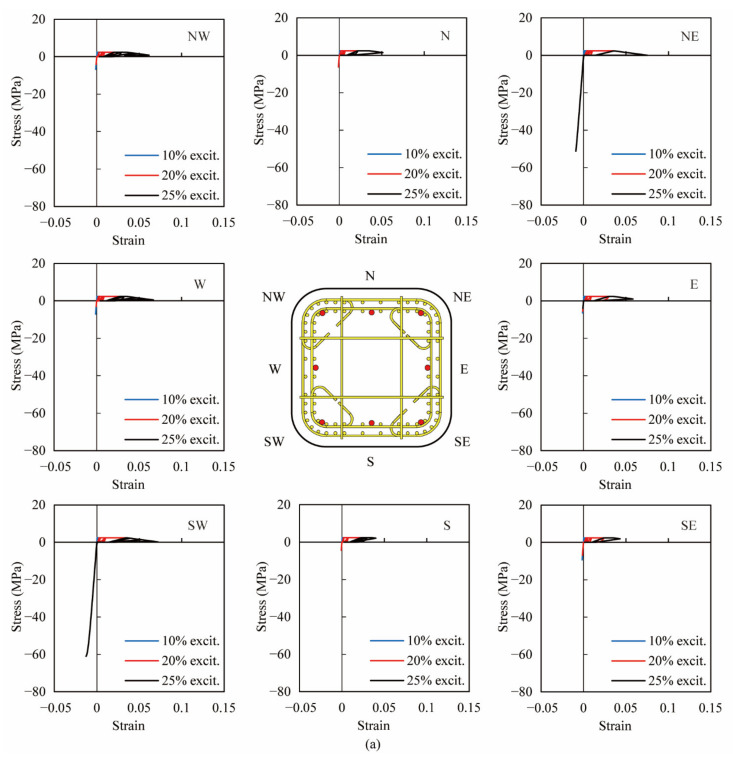

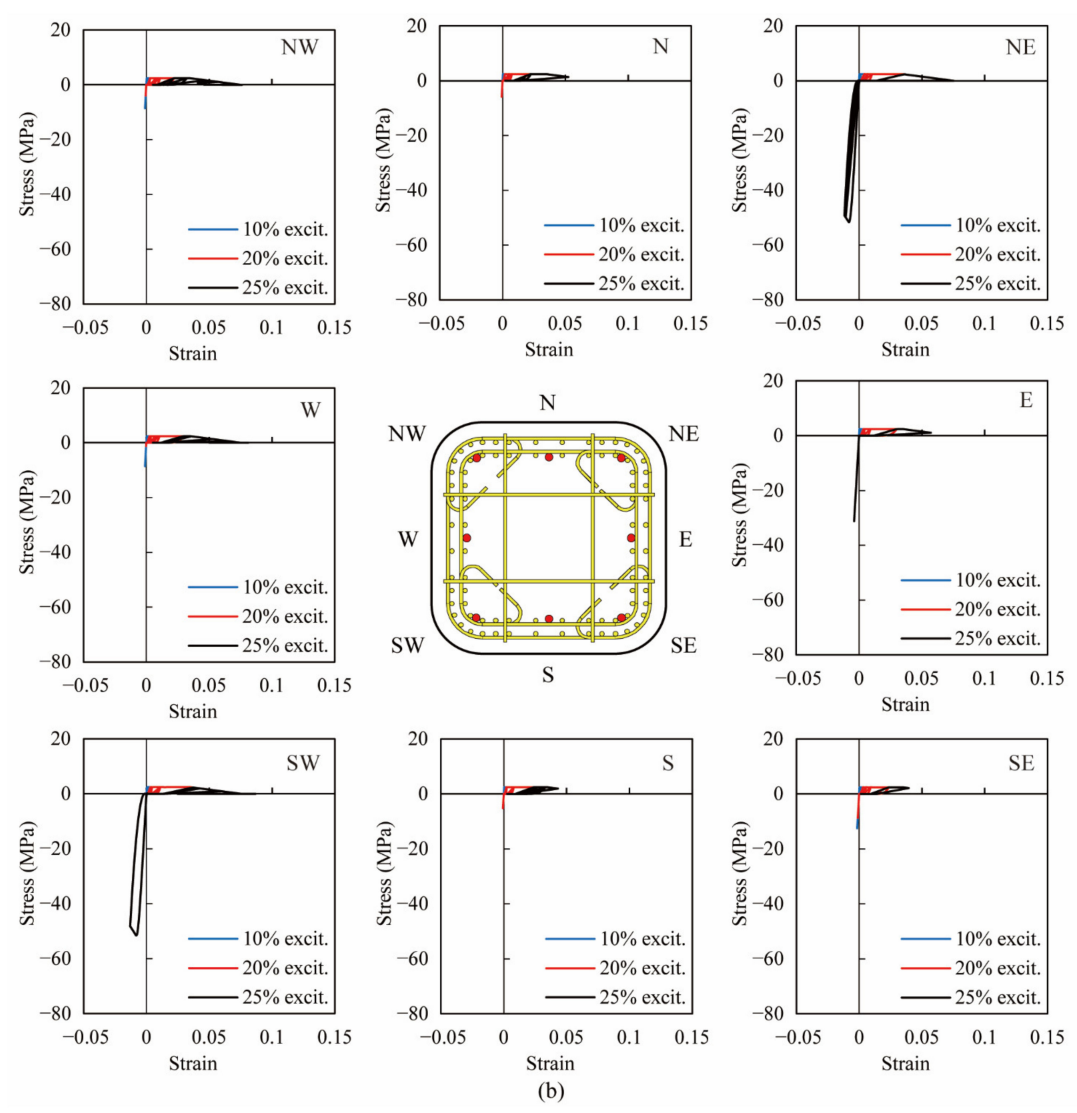
Analytical stress-strain hysteresis of extreme fibers in core at the column base in eight directions. (**a**) PFRC-1, (**b**) PFRC-2.

As shown in Figure 9, Figure 10 and Figure 11, due to the analytical results being in good agreement with the experiments, the analytical stress-strain hysteresis of extreme fibers in the cover and core concrete was used to clarify the effect of the volume tie ratio. Figure 12 and Figure 13 show the analytical stress-stain hysteresis of extreme fibers of the cover and core ECC in PFRC-1 and 2 columns at the column base during all excitations, respectively. Hereinafter, ‘excitation’ is referred to as ‘excit’. The locations of extreme fibers in eight directions, namely, East (E), South (S), West (W), North (N), Northeast (NE), Northwest (NW), Southwest (SW) and Southeast (SE), in the cross sections were illustrated by the red dots in the central sub-figure of Figure 12 and Figure 13. As shown in Figure 12, all fibers underwent large nonlinear deformation in tension, and stress-strain hysteresis of these fibers went into the strain-softening phase, indicating that the localized cracks at the column base were developed and joined up. Moreover, the compressive behavior of the fibers in the E, NE and SW direction during the 3rd excitation in both columns was more significant than the rest of the five directions, showing that the crushing of the ECC cover in these three directions occurred in both columns. The analytical result was consistent with experimental phenomena. By comparing the stress-strain hysteresis of the ECC cover in PFRC-1 with that in PFRC-2, little difference could be observed, revealing that the volume tie ratio had little effect on the ECC cover.

Similar to Figure 12, the fibers in all directions at the edge of the core also went into a tension-softening phase on the tension side as shown in Figure 13, indicating that the localized cracks were also propagated into the edge of the core at the column base. The compressive behavior of fibers in the NE and SW direction was more significant. In PFRC-1, the peak stress of fibers at the NE and SW was still within the compressive strength of the double-confined ECC. However, in PFRC-2, the stress-strain hysteresis of fibers in the NE and SW were involved in the nonlinear behavior due to smaller compressive strength in the core.

### 4.2. Hysteresis of Stress-Strain in Longitudinal Bars

**Figure 14:**
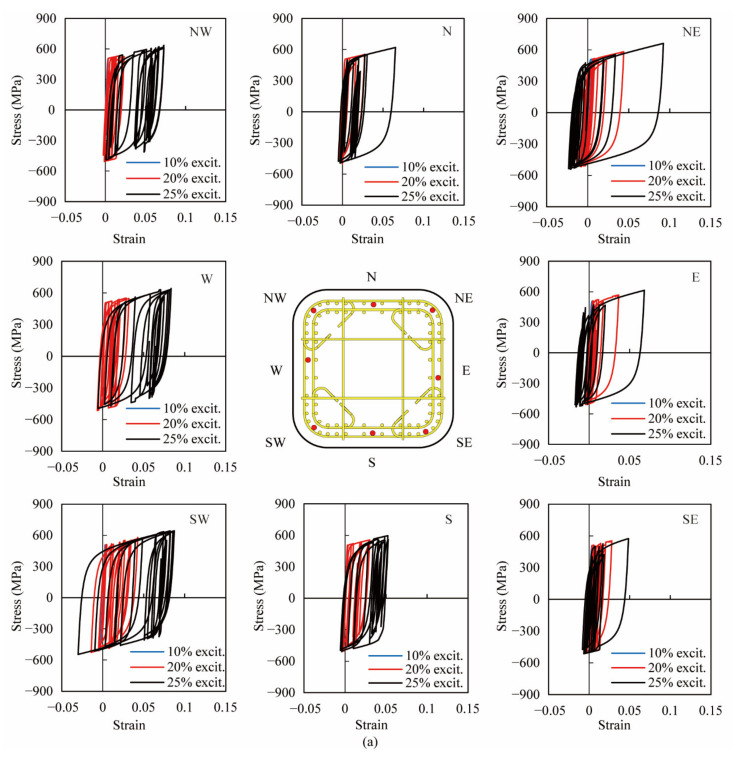

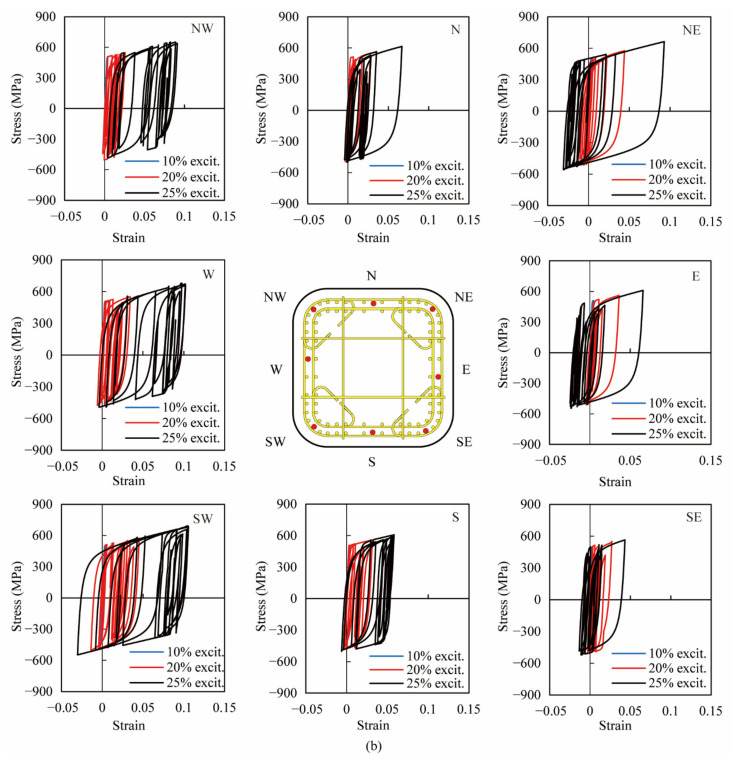
Analytical hysteresis of stress-strain in longitudinal bars at the column base. (**a**) PFRC-1, (**b**) PFRC-2.

The hysteresis of stress-strain in the longitudinal bars at the column base in two columns was also investigated based on the analytical results. Figure 14 shows the analytical hysteresis of stress-strain of the longitudinal bars in eight directions as illustrated by red dots in the central sub-figure. The analytical stress-strain hysteresis of the longitudinal bars in eight directions was still within the elastic range during the 1st excitation, while they were involved in nonlinearity during the 2nd and 3rd excitation and still within the rupture strain of 0.23. The nonlinear behavior of the longitudinal bars in the NE and SW direction was more significant than the rest of the bars in both columns. Stress-strain hysteresis in two columns was almost identical to each other during the 1st and 2nd excitation. It indicated that ECC could reduce the local buckling in longitudinal bars when the column with the reduction in ties underwent moderate nonlinear deformation, due to its superior ductility and damage tolerance. During the 3rd excitation, the nonlinear strain of longitudinal bars in the NW, W, SW and S directions in PFRC-2 was larger than that in PFRC-1, which was consistent with the larger response displacement in the W direction in the experiment. When the column was subjected to the large nonlinear deformation, the necessary lateral confinement to the longitudinal bars was beyond the capacity of ECC, and more ties were still essential to prevent residual deformation.

### 4.3. Maximum Allowable Displacement for Design

**Figure 15:**
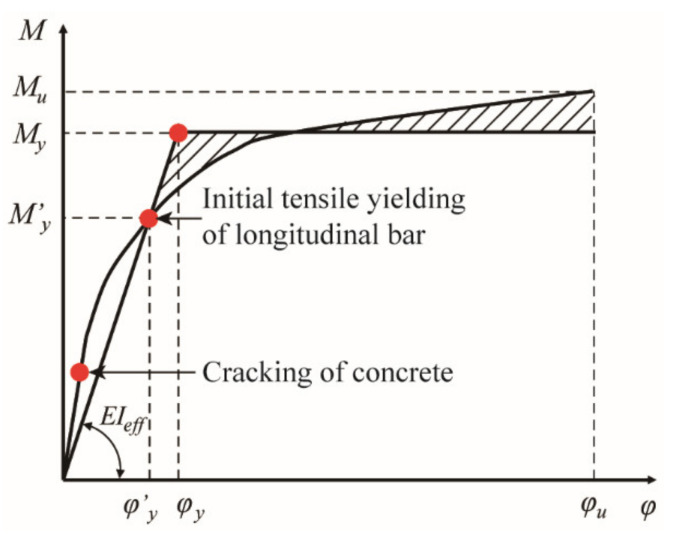
Equivalent yielding moment and curvature.

The displacement-based seismic design method is currently widely adopted by design codes in many countries, in which maximum allowable displacement is one of the most important design parameters. In this study, the maximum allowable displacement ($\Delta_{u}$) is calculated based on the equivalent yielding curvature as described in Chinese code [24], and it is expressed by Equation (15):

(15) $$\begin{array}{c} \Delta_{u}=\frac{1}{3}H^{2}\phi_{y}+\left(H-\frac{L_{p}}{2}\right)\theta_{u} \end{array}\;$$

where H is the height of the cantilever column (cm); $\phi_{y}$ is the equivalent yielding curvature and can be determined according to the moment versus curvature relationship as shown in Figure 15; $L_{p}$ is the length of the plastic hinge (cm) and is defined by Equation (16) in Chinese code [24]; $\theta_{u}$ is the maximum allowable rotation angle in the region of the plastic hinge and can be determined by Equation (17).

(16) $$\begin{array}{c} L_{p}=\min\left(0.08H+0.022f_{y}d_{s}\geq 0.044f_{y}d_{s},\frac{2}{3}\right) \end{array}$$

(17) $$\begin{array}{c} \theta_{u}=L_{p}\left(\phi_{u}-\phi_{y}\right)/K \end{array}$$

where $f_{y}$ is the characteristic value of tensile yield strength (MPa); $d_{s}$ is the diameter of the longitudinal bar (cm); b is the shorter side length of the rectangular or diameter of the circular section (cm). $\phi_{u}$ is the curvature at the limit state (1/m); K is the safety factor for ductility and is taken as 2.0.

**Figure 16:**
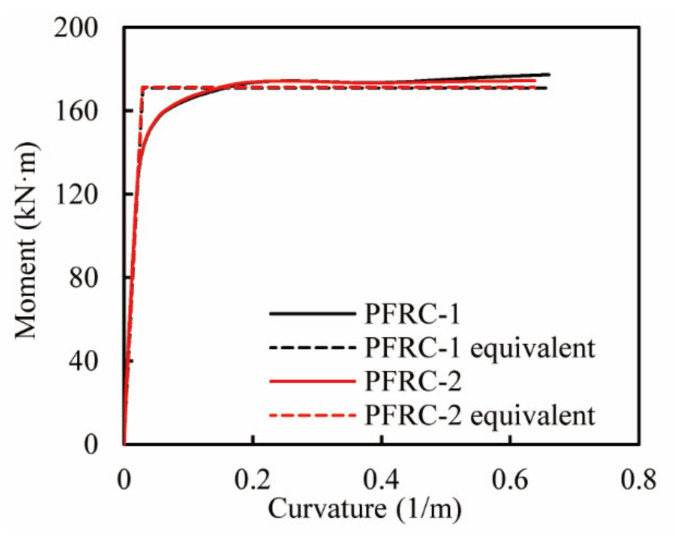
Equivalent yielding moment and curvature at column base.

To clarify the effect of the volume tie ratio on the maximum allowable displacement, a pushover analysis was conducted by applying a vertical constant load of 86.4 kN and a lateral monotonic displacement load. The analytical results of the moment versus curvature relationship at the column base in both columns are shown in Figure 16 by using solid lines. The equivalent yielding moment versus curvature in both columns was shown with dotted lines. There is little difference in the analytical moment versus curvature relationship between the two columns before aa curvature of 0.4, while a slight difference can be observed after a curvature of 0.4. Meanwhile, the equivalent yielding moment and curvature in both columns are almost the same. Consequently, the equivalent yielding moment and curvature in both columns are tabulated in Table 5. Thereby, the maximum allowable displacement for the design can be calculated as listed in Table 5. $\Delta_{u}$ in PFRC-1 is larger than that in PFRC-2 by 3.4 mm, indicating that the effect of the volume tie ratio in the ECC plastic hinge results in a limit increase in the maximum allowable displacement for the design.

## 5. Conclusions

To investigate the effects of the volume tie ratio in ECC plastic hinges on the seismic performance of RC composite bridge columns, the numerical study on the seismic performance of the RC bridge columns by using ECC in the region of the plastic hinge with a varying volume tie ratio was conducted based on hybrid loading experiments. Based on the results presented, the following conclusions could be drawn:

(1) The proposed calculation for the lateral confining stress of the ECC cover was verified to be valid due to the analytical results being in good agreement with the experiments based on the fiber-element-based analysis. It also indicates that the reduction in the volume tie ratio has little effect on the hysteresis of lateral force displacement because the ECC cover has already provided substantial lateral confinement.
(2) The reduction in the volume tie ratio has little effect on the stress-strain hysteresis of the ECC cover, but it has an effect on the stress-strain hysteresis of core ECC due to different compressive strengths in core ECC resulting from a different extent of lateral confinement. With the reduction in the volume tie ratio, the ECC cover can reduce the local buckling in longitudinal bars only if the column undergoes small or moderate nonlinear deformation. Once the deformation was large, more ties were still essential to provide necessary lateral confinement to mitigate local buckling of the longitudinal bars.
(3) Based on the theory of equivalent yielding curvature, the volume tie ratio in ECC plastic hinges has a limited effect on the maximum allowable displacement for design. The maximum allowable displacement of the composite column with a volume tie ratio of 1.51% is larger than that with a volume tie ratio of 0.76% by 3.4 mm. It indicates that the increase in the volume tie ratio in the plastic hinge region of the ECC column is no longer significantly effective in enhancing the ductility of the column. It is possible to reduce the quantity of ties in the plastic hinge region and therefore to reduce the difficulty in placing concrete during the construction phase and prevent potential placing defects and future durability problems.

## Figures and Tables

**Table 1:** Mix proportion of ECC [10].

W/B ^1^ (%)	FA/B ^2^ (%)	Unit Weight (kg/m^3^)		
		W	B	PP ^3^
27	33	371	1400	27

^1^ stands for water-to-binder ratio; ^2^ stands for fly ash to binder ratio; ^3^ stands for polypropylene fiber.

**Table 2:** Similitude relation in design phase.

Physical Quantity	Similitude Relation	Similitude Parameter
Length L	$S_{L}$	6/35
Displacement Δ	$S_{\Delta }=S_{L}$	6/35
Elastic modulus E	$S_{E}=E_{m}∕E_{p}$	1
Stress σ	$S_{\sigma }=S_{E}$	1
Force F	$S_{F}=S_{\sigma }S_{L}S_{L}$	36/1225

Note: $E_{m}$ and $E_{p}\;$are the elastic modulus of the model and prototype, respectively.

**Table 3:** Parameters in unconfined and confined ECC models.

Parameters	Unconfined ECC	Double-Confined ECC		Triple-Confined ECC	
		PFRC-1	PFRC-2	PFRC-1	PFRC-2
$\mathrm{Compressive}\;\mathrm{strength}\;f_{ECC}^{\prime }$ (MPa)	45.05	51.03	47.69	61.05	51.66
$\mathrm{Strain}\;\mathrm{at}\;\mathrm{compressive}\;\mathrm{strength}\;\varepsilon_{ECC}^{\prime }$	0.0047	0.0078	0.0061	0.013	0.0081
$\mathrm{Ultimate}\;\mathrm{compressive}\;\mathrm{strain}\;\varepsilon_{u\_ECC}^{\prime }$	0.047				
$\mathrm{Tensile}\;\mathrm{cracking}\;\mathrm{stress}\;f_{cr\_ECC}$ (MPa)	2.4				
$\mathrm{Strain}\;\mathrm{at}\;\mathrm{tensile}\;\mathrm{cracking}\;\mathrm{stress}\;\varepsilon_{cr\_ECC}$	0.000293				
$\mathrm{Ultimate}\;\mathrm{tensile}\;\mathrm{stress}\;f_{tu\_ECC}$ (MPa)	2.4				
$\mathrm{Strain}\;\mathrm{at}\;\mathrm{ultimate}\;\mathrm{tensile}\;\mathrm{stress}\;\varepsilon_{tu\_ECC}$	0.035				

**Table 4:** Parameters in Steel model.

Properties	PFRC-1	PFRC-2
$\mathrm{Yield}\;\mathrm{stress}\;\mathrm{in}\;\mathrm{tension}\;f_{y}$ (MPa)	507	
Initial elastic tangent E (MPa)	182,000	
Ratio between post-yield tangent and initial elastic tangent	0.01	
Parameter to control the transition from elastic to plastic branches	18	

**Table 5:** Displacement-based seismic design parameters.

**Column**	$M_{y}^{\prime }$ (kN·m)	$M_{y}$ (kN·m)	$\phi_{y}\;$ (1/m)	$M_{u}$ (kN·m)	$\phi_{u}\;$ (1/m)	$\phi_{u}-\phi_{y}$ (1/m)	$\theta_{u}$	$L_{p}$ (m)	$\Delta_{u}$ (mm)
PFRC-1	127.84	170.98	0.0289	177.29	0.6637	0.6348	0.06348	0.2	98.7
PFRC-2	127.87	171.39	0.0284	174.31	0.6387	0.6103	0.06103	0.2	95.3

Note: $M_{y}^{\prime }$ is the initial yielding moment; $M_{y}$ is the equivalent yielding moment; $\phi_{y}$ is the equivalent yielding curvature; $M_{u}$ is the ultimate moment.

## Data Availability

Data are available on request from the authors.
